# Modified toothpaste application using prepared toothpaste delivering technique increases interproximal fluoride toothpaste delivery

**DOI:** 10.1002/cre2.268

**Published:** 2019-12-12

**Authors:** Ryouichi Satou, Seitaro Suzuki, Atsushi Takayanagi, Atsushi Yamagishi, Naoki Sugihara

**Affiliations:** ^1^ Department of Epidemiology and Public Health Tokyo Dental College Tokyo Japan

**Keywords:** brushing, fluoride, fluoride delivery, fluoride uptake, toothpaste technique

## Abstract

**Objectives:**

We devised a “prepared toothpaste delivering technique” (PTD technique), a modified the application of toothpaste method for using fluoride toothpaste more effectively. This study aimed to investigate the change in viscosity and fluoride intake into hydroxyapatite of a toothpaste, and deliverability of fluoride toothpaste to the interproximal site with the PTD technique using an interproximal model.

**Methods:**

Eight toothpaste samples were prepared at the following concentrations: ×1.00, ×1.25, ×1.50, ×1.75, ×2.00, ×3.00, ×4.00, and ×5.00. Viscosity of the toothpaste was measured by a Type‐B viscometer. Dissolution rate of toothpaste and fluoride uptake into the hydroxy apatite pellet were analyzed by a fluoride selective electrode. Application paste volume and delivery rate was measured using interproximal model and image analysis software during using a finger brush front (FBF), finger brush back (FBB), and toothbrush.

**Results:**

As the dilution ratio increased, the viscosity of the toothpaste decreased sharply, *F* uptake decreased, and dissolution rate increased. *F* uptake was significantly reduced when the toothpaste was diluted more than 1.75 times. Therefore, in order to improve the effectiveness of the fluoride toothpaste, it is important to deliver the toothpaste to interproximal areas and pit clefts at low dilution. It was observed that PTD technique can be effectively implemented by the finger brush.

**Conclusions:**

The use of a FBF surface in the analysis of an acrylic interproximal model could aid in applying pressure while blocking the space of the groove and preventing outflow of the toothpaste. It was considered that the PTD technique would improve the effects of the fluoride toothpaste, especially in the interproximal site.

## 1 INTRODUCTION

Prevalence of dental caries has decreased in developed countries (Selwitz, Ismail, & Pitts, 2007). In a systematic literature review, Frencken et al. reported that the prevalence and severity of dental caries among 5 and 12 years olds have declined over the last decades (Frencken et al., 2017). This phenomenon is reported to be significantly associated with the widespread use of fluoride (F^−^) toothpaste (Walsh, Worthington, Glenny, Marinho, & Jeroncic, 2019). It is known that a toothpaste with higher fluoride concentration has a greater effect in preventing dental caries (Mannaa et al., 2014; Walsh et al., 2019). Although the prevalence of dental caries has decreased, the interproximal surface is still a high‐risk site of dental caries (Haak, Wicht, Hellmich, Gossmann, & Noack, 2002). However, it is difficult for the bristles of the toothbrush to reach this site (Kiger, Nylund, & Feller, 1991).

A modified toothpaste technique advocated by K. Sjögren in which a slurry rinse with a fluoride toothpaste is used after brushing increases the efficacy of the fluoride toothpaste (Sjögren, 1995). Moreover, this toothpaste technique has been reported to reduce interproximal caries in preschool children by an average of 26% (Sjögren, Birkhed, & Rangmar, 1995). The purpose of using this toothpaste technique is delivering fluoride to sites with high risk of dental caries. Tooth paste is diluted by saliva secreted during tooth brushing, and this slurry rinse is effective for delivering fluoride to the high‐risk site (Sjögren, 1995). Although the diluted toothpaste has high deliverability, it is reasonable to assume that the concentration of fluoride will decrease and the adherence to the surface of the teeth will reduce. Meanwhile, post‐brushing rinsing behavior has been reported to be associated the anticaries effect provided by fluoride toothpaste (Duckworth et al., 2009; Parnell & O'Mullane, 2013). Duckworth et al. reported that rinsing for 5 s with 10 mL water and rinsing for 30 s with 20 mL of NaF mouthwash were effective for maintaining a high fluoride concentration in the saliva compared with rinsing for 30 s with a non‐fluoridated mouthwash (Duckworth et al., 2009). In addition, avoiding or minimizing rinsing with water is recommended by several guidelines for caries prevention (Parnell & O'Mullane, 2013). Therefore, maintenance of a certain level of fluoride in the mouth can be considered a significant factor for caries prevention.

There has been no quantitative study of the relationship between *F* uptake and the viscosity and persistence of toothpaste, which change with dilution. Changes in the physical properties and functions of the tooth paste that occur over time in the oral cavity should be examined, and development of a method to improve the effect of fluoride on carious sites is required.

Therefore, we devised a “prepared toothpaste delivering technique” (PTD technique). This technique uses a finger brush made of silicone rubber, before tooth brushing, to deliver the toothpaste to a high‐risk site without decrease in the concentration of fluoride due to dilution of saliva. The PTD technique consists of two phases, the “application phase” and the “brushing phase,” as in the experiment. In the application phase, 1.0 g of toothpaste is set on the toothbrush, and the undiluted toothpaste is applied to all the teeth, starting from the interproximal space of the molar region. In the brushing phase, brushing is performed at 200 g pressure for about 3 min using a toothbrush. At the time of brushing, it is preferable to brush at an angle of 15°–30° from the vertical to application toothpaste to the tooth surface so that the brush tuft tips can be easily inserted into the interproximal region. The toothpaste is delivered to the interproximal region in a highly viscous state, and there is no need to limit rinsing.

In modified toothpaste technique, rinsing with a small amount of water is recommended to deliver fluoride to the interproximal region in the form of a slurry (Sjögren, 1995). However, since the PTD method does not require water restriction, it is considered to be a method that has a relatively high degree of freedom and is easy to practice on a daily basis. In addition, the difficulty of the PTD technique is low because the finger is used directly, and it can be practiced not only by adults but also by young children and elderly people.

The purpose of this study was to investigate the change in viscosity and fluoride intake into hydroxyapatite of a toothpaste. Moreover, we investigated the deliverability of fluoride toothpaste to the interproximal site with the PTD technique using an interproximal model. The hypothesis of the present study was that the PTD technique would improve the effects of fluoride toothpaste, especially in the interproximal sites.

## 2 STUDY POPULATION AND METHODOLOGY

### 2.1 Experimental design

This study was consisted of two in vitro model experiments. In Experiment 1, the relationship between *F* uptake and viscosity and durability of toothpaste, which changes with dilution, was investigated quantitatively. In Experiment 2, the toothpaste delivery rate to the interproximal area of dental model was investigated by using the PTD technique. On the basis of the results of these two experiments, we propose a new method to deliver fluoride more effectively to the interproximal area by clarifying the changes in the physical properties and function of toothpaste that occur over time in the oral cavity.

### 2.2 Preparation of toothpaste and dental instrument

The toothpaste used in this study was Clear Clean Double Plus (KAO Corp., Tokyo, Japan), with 950 ppm *F* in the form of sodium fluoride (NaF), which is commonly available in Japan. The toothpaste was diluted stepwise with ion‐exchanged water for studying the viscosity and decline rates. Eight samples were prepared at the following concentrations: ×1.00, ×1.25, ×1.50, ×1.75, ×2.00, ×3.00, ×4.00, and ×5.00. In this experiment, a commercially available general GUM dental brush #211 toothbrush (3‐row compact head, normal, GUM Corp., Tokyo, Japan) was used. The device used in the “application phase” for delivery of the toothpaste to the interproximal area is made of a material that adheres to the fingers and is soft and elastic, finger brush (Deep Clean, M size, KAO Corp.) (Figure S1). This product is made of silicone and is shaped like a finger cap, which is composed of two surfaces: a front (FBF) surface with multiple small projections and a flat back (FBB).

### 2.3 Operators

Five right‐handed dentists of Tokyo Dental College familiar with the use of tooth brush performed the experiments. The operators had sufficient experience with brushing in an interproximal model. The brushing methods were calibrated between operators.

### 2.4 Determination of fluoride concentration

All samples were analyzed for F^−^ concentration by a fluoride combination ion selective electrode (9609BNWP, Thermo Science, Waltham, MA) and an ion meter (930A, Thermo Science). The standard fluoride concentrations were 10, 1.0, and 0.1 ppm, and the solutions were prepared by dissolving NaF in the mixture of TISAB III at the ratio of 1–10. Fluoride concentrations were calculated by comparison with a standard curve using three standards ranging from 0.1 to 10 ppm standard.

### 2.5 Fluoride uptake into the hydroxy apatite pellet

Hydroxy apatite pellet (HAp, Apaceram APP‐100, 10 × 10 × 2 mm, HOYA Technosurgical Corp., Tochigi, Japan) was used as a model of enamel. The HAp pellet was mirror‐polished with water resistant abrasive paper (C#1000, Coated Abrasive and Imperial wrapping film, 12 μm, 9 μm, 3 M Japan, Tokyo, Japan). A window measuring 10 × 10 mm was made using a nail varnish (RD‐61 coffret dor, Kanebo, Tokyo, Japan). After application of eight concentration samples (×1.0, ×1.25, ×1.50, ×1.75, ×2.00, ×3.00, ×4.00, and ×5.00) to the window for 3 min, the pellets were washed out with ion‐exchanged water for 30 s and dried. After immersion in 0.5 ml of 1 M hydrochloric acid for 30 s, quantification was performed using ion electrode.

### 2.6 Viscosity of toothpaste

Viscosity of the toothpaste was measured by a Type‐B viscometer (TVB‐10, TOKI SANGYO, Tokyo, Japan). The appropriate rotor and number of revolutions were selected according to the toothpaste dilution (H7 rotor, 2.5 rpm for ×1.0, ×1.25; H5 rotor, 2.5 rpm for ×1.5; H2 rotor, 2.5 rpm for ×1.75; H2 rotor, 1.0 rpm for ×2.00, ×3.00, ×4.00, ×5.00).

### 2.7 Dissolution rate of toothpaste

There have been no studies on the removal of dentifrice adhering to the tooth surface, and a measurement method was developed in this study. Fifty milligrams of the toothpaste was applied to a one hole slide glass (2‐149‐01, hole 14–15 mm, ASONE, Tokyo, Japan). A 5‐L beaker was charged with 4‐L of ion exchange water and attached to a stirring blade. Since the undiluted dentifrice remained for a long time even when immersed in water, a water flow was generated for efficient evaluation. It took a long time at a low flow rate, and turbulence occurred at a high flow rate and the experiment became unstable. Therefore, the flow rate was adjusted to be as fast as possible within the range where stable experiments were possible. Therefore, the toothpaste flowed out was adjusted to a speed at which the all amount of toothpaste (×1.00) was washed out in 150 s. The slide glass was held in water at a depth of 1 cm below and parallel to the water surface, and the residual amount of toothpaste at 15, 30, and 60 s was measured (Figure S2). The residual amount of toothpaste was determined by measuring the fluoride concentration of the dissolved water. The dilution rate (mg/s) was calculated from the residual amount of toothpaste at each dilution concentration at 30 s.

### 2.8 Production of acrylic interproximal model

An acrylic interproximal model was created for this experiment. Black acrylic round bars (MMB‐BH‐1002, Black, diameter 8 mm, Hazaiya, Tokyo, Japan) were cut to a length of 8 cm, line up 14 horizontally aligned and adhered, and fixed to the base (Figure S3). The model has 13 interproximal areas, which are 4 mm deep. The color of acrylic was black to emphasize the contrast with the white toothpaste.

### 2.9 Measuring delivery rate and application paste volume of toothpaste with the acrylic interproximal model

The experiment consists of an (a) “application phase” for delivery of toothpaste to the interproximal region and a (b) “brushing phase” for diffusion and cleaning of the toothpaste. In order to examine toothpaste delivery, three methods were established with different instruments used in the application phase: (a) applying toothpaste with a finger brush front (FBF) in the application phase and followed by brushing with a toothbrush; (b) applying toothpaste with a finger brush back (FBB) in the application phase and followed by brushing with a toothbrush; and (c) both the application phase and the brushing phase are performed using only a toothbrush. We defined FBF and FBB as the experimental group and only the toothbrush as the control group. The amount of toothpaste used in application phase was 1.0 g, the brushing pressure in the brushing phase was 200 g, and the movable range was the entire area of the acrylic interproximal model. Several cycles brushing were carried out until the dentifrice no longer enters the gap. The brush was applied perpendicular to the model surface only in brushing phase. After the end of each phase, the weight of the toothpaste remaining on the brush was measured, and the amount of toothpaste delivered was calculated according to the following equation:

$$\text{Application paste volume}\left(\%\right)=\frac{\text{Weight}\;\mathrm{at}\;\text{start}\;\left(1.0\;g\right)-\text{brush remaining weight}}{\text{Toothpaste weight}\;\mathrm{at}\;\text{start}\;\left(1.0\;g\right)}\times 100$$

### 2.10 Image analysis for measuring the delivery rate

In order to examine toothpaste deliver rate, five methods were established with different instruments used (a) applying toothpaste with a FBF; (b) FBF and followed by brushing with a toothbrush (FBF + TB); (c) applying toothpaste with a FBB; (d) FBB and followed by brushing with a toothbrush (FBB + TB); and (e) using only a toothbrush (TB). At the end of each phase, images were taken by a digital camera (ILCE‐7M3, Sony, Tokyo, Japan) with a micro lens (AI Micro‐Nikkor 55 mm f/2.8S, Nikon, Tokyo, Japan) vertically 15 cm above the acrylic interproximal model. Images were taken to analyze the length of the toothpaste delivered to the interproximal area. All images were taken with a linear polarizing filter attached to the lens and the imaging light to avoid reflections of the acrylic model surface. Of the toothpaste remaining in the interproximal model, the length of the toothpaste filling the interproximal groove was measured using image analysis software (Image J, version 1.52) (Abramoff, Magalhães, & Ram, 2004; Schneider, Rasband, & Eliceiri, 2012). Delivery rate was calculated based on the following equation:

$$\text{Delivery rate}\;\left(\%\right)=\frac{\sum \left[\text{Each paste length}\;\left(\mathrm{mm},\text{line}\;1\;\text{to}\;13\right)\right]}{\text{Total interdental length}\;\left(\mathrm{mm},80\;\mathrm{mm}\times 13\;\text{line}\right)}\times 100$$

### 2.11 Statistical analysis

In experiment on fluoride uptake and viscosity of toothpaste, the statistical analysis among the test groups was performed by one‐way analysis of variance (ANOVA), and differences were considered significant at *p* < .05. Data in each group are presented as mean ± *SD* of five replicates (*n* = 5). The Bonferroni test was used for post‐hoc comparisons when significance was determined by analysis of variance (*p* < .05).

The application paste volume and delivery rate of the toothpaste are presented as mean ± *SD* of five replicates per brushing method (*n* = 5). *p* Values were calculated by one‐way ANOVA and results were considered significant at *p* < .001. The Bonferroni test was used for post‐hoc comparisons when significance was determined by analysis of variance (*p* < .001).

## 3 RESULTS

### 3.1 Fluoride uptake into the HAp pellet according to dilution rate of toothpaste

**Figure 1:**
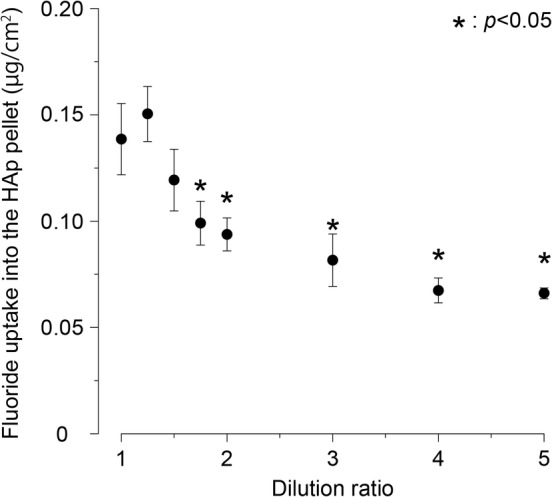
Effect of fluoride uptake into HAp pellet surface according to dilution ratio of toothpaste. The fluoride uptake concentrations are presented as mean ± *SD* of five replicates per dilution ratio (*n* = 5). *p* Values were calculated by one‐way ANOVA, and results were considered significant at *p* < .05. The Bonferroni test was used for post‐hoc comparisons when significance was determined according to the sample (×1) by analysis of variance (*p < .05). It was significantly reduced at dilutions of ×1.75, ×2.00, ×3.00, ×4.00, ×5.00 compared with the original solution (×1.00) (*p < .05). The fluoride uptake tended to decrease with dilution. HAp, hydroxy apatite

Fluoride uptake into the HAp pellet was high in the original (×1.00) and ×1.25 solution. It was significantly reduced at dilutions of ×1.75 or more compared with the original solution (×1.00) (*p < .05) (Figure 1). The fluoride uptake tended to decrease with dilution. At ×5.00 dilution, it was reduced to about half of that of the original solution (×1.00) (Figure 1 and Table 1). The uptake at ×1.25 dilution tended to be higher than that in the original solution (×1.00), but there was no significant difference from the stock solution (Figure 1). The uptake at a dilution of ×2.00 or more showed less fluctuation compared with ×1.00–×1.75 dilution. Fluoride uptake was nearly constant at ×2.00–×5.00 dilution (Figure 1).

**Table 1:** Relationship between the dissolution rate and dilution rate of the toothpaste

Dilution ratio	Residual amount of toothpaste (mg)			Dissolution (mg/s)
	15 s	30 s	60 s	(during 0–30 s)
×1.00	31.20 ± 0.07	24.56 ± 2.16	11.40 ± 2.86	0.88 ± 0.09
×1.25	29.87 ± 2.59	19.68 ± 3.81	5.22 ± 3.13	0.95 ± 0.12
×1.50	30.60 ± 2.02	14.53 ± 5.63	—	1.23 ± 0.18
×1.75	23.86 ± 1.62	9.58 ± 2.23	—	1.35 ± 0.05
×2.00	4.34 ± 1.93	0.12 ± 0.02	—	1.69 ± 0.02
×3.00	—	—	—	>1.67
×4.00	—	—	—	>1.67
×5.00	—	—	—	>1.67

### 3.2 Change in toothpaste viscosity according to dilution rate

**Figure 2:**
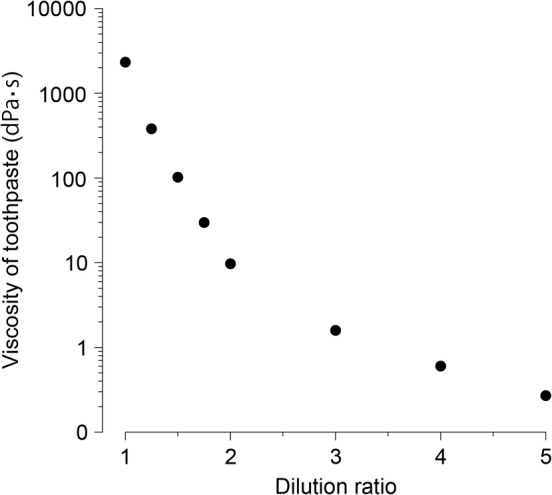
Effect of viscosity according to dilution ratio of toothpaste. Viscosity of toothpaste is plotted as log by dilution ratio. The viscosity decreased rapidly upon dilution

The viscosity was the highest in the original solution (×1.00) and decreased rapidly with the dilution ratio (Figure 2). Compared with the stock solution, the viscosity was about 1/5 at ×1.25 dilution, about 1/20 at ×1.5 dilution, and about 1/100 at ×1.75 dilution. The viscosity reduction was remarkable up to ×1.75, and it became moderate and nearly constant at dilution ratios of ×2.00 and higher. When diluted to ×5.00, viscosity decreased to about 1/800 compared to the original solution (×1.00). The viscosity of ×5.00 decreased to almost the same as the viscosity of pure water.

### 3.3 Change in dissolution rate according to toothpaste dilution

The dissolution rate was increased by dilution, and samples of ×3.00 or more dilution (×3.00, ×4.00, and ×5.00) were washed out within 15 s (Table 1). In the case of 30 s, the residual amount of the original solution (×1.00) and the solution with ×1.25 dilution also decreased compared with 15 s. The dissolution rate calculated based on the data for 30 s was ~1.15 times at ×1.25, ~1.40 times at ×1.50, ~1.57 times at ×1.75, and approximate double when the dilution ratio was ×2.00 (Table 1). In the case of 60 s, all dilutions except the original solution (×1.00) and the solution with ×1.25 dilution were washed away. “Expected effect index (F/D)” from the relationship between fluoride uptake (*F*) and dissolution rate (*D*) (Table 2). It decreased as the dilution ratio increased, and the original solution (×1.00) showed the highest value (Table 2).

**Table 2:** Expected effect index of the dilution rate of the toothpaste

Dilution ratio	Fluoride uptake (μg/cm^2^)	Dissolution (mg/s) (during 0–30 s)	Expected effect index
×1.00	0.14 ± 0.02	0.88 ± 0.09	0.159
×1.25	0.15 ± 0.01	0.95 ± 0.12	0.158
×1.50	0.12 ± 0.01	1.23 ± 0.18	0.100
×1.75	0.10 ± 0.01	1.35 ± 0.05	0.073
×2.00	0.09 ± 0.01	1.69 ± 0.02	0.055

*Note:* Expected effect index = Fluoride uptake (*F*)/Dissolution speed (during 0–30 s) (*D*).

### 3.4 Percentage of application paste volume using PTD technique

**Figure 3:**
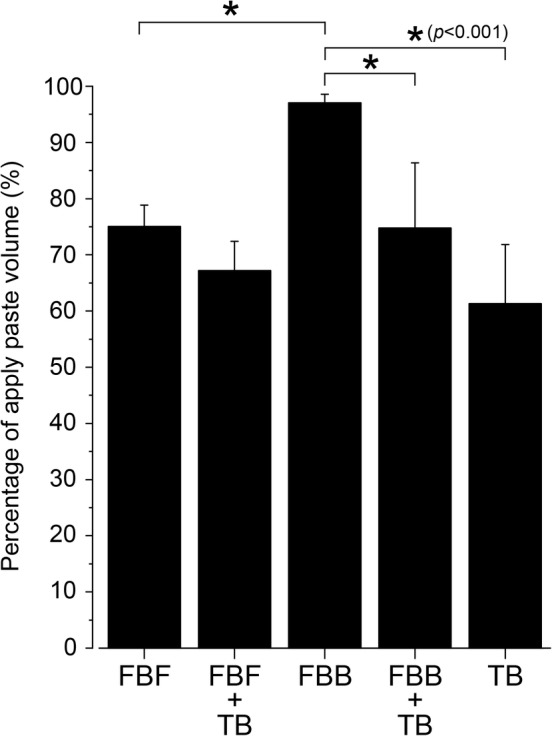
Application paste volume of toothpaste on an interproximal model using the brushing method. The application paste volume of the toothpaste is presented as mean ± *SD* of five replicates per brushing method (*n* = 5). FBF, finger brush front, a front with multiple small silicone projections; FBB, finger brush back, flat surface; TB, toothbrush only; FBF + TB, the application phase was performed at the front (FBF) and the brushing phase was performed with a toothbrush (TB); FBB + TB, the application phase was performed at the back (FBB) and the brushing phase was performed with a toothbrush (TB). *p* Values were calculated by one‐way ANOVA and results were considered significant at *p* < .001. The Bonferroni test was used for post‐hoc comparisons when significance was determined by analysis of variance (*p < .001). The amount of toothpaste applied to the interproximal area was highest in the FBB group among the three groups and was significantly increased in the FBB compared to the TB (*p* < .001). In addition, compared to the applied amount of 75% for FBF, an increase in the applied amount of 95% was observed for FBB (*p* < .001)

The amount of toothpaste applied to the interproximal area was highest in the FBB group among the three groups and was significantly increased in the FBB compared to the TB (*p* < .001) (Figure 3). There was no significant difference between TB and FBF. In addition, compared to the applied amount of 75% for FBF, an increase in the applied amount of 95% was observed for FBB (*p* < .001) (Figure 3). When comparing the FBF group with the FBF + TB group subjected to the brushing phase, a decrease in the applied amount by about 10% was observed, but there was no significant difference. In contrast, when the FBB group was similarly compared with the FBB + TB group, a sharp drop in the applied amount by about 20% was observed (*p* < .001).

### 3.5 Delivery rate of toothpaste by using the PTD technique

**Figure 4:**
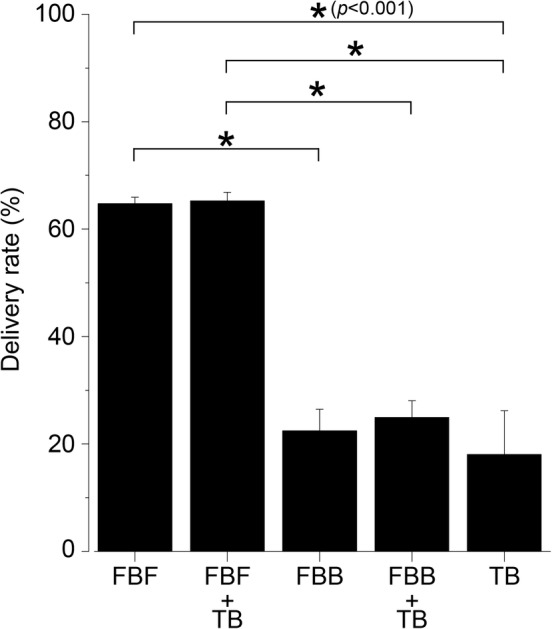
Delivery rate of toothpaste in the interproximal area. The application paste volume of the toothpaste is presented as mean ± *SD* of five replicates per brushing method (*n* = 5). FBF, finger brush front, a front with multiple small silicone projections; FBB, finger brush back, flat surface; TB, toothbrush only; FBF + TB, the application phase was performed at the front (FBF) and the brushing phase was perform ed with a toothbrush (TB); FBB + TB, the application phase was performed at the back (FBB) and the brushing phase was performed with a toothbrush (TB). *p* Values were calculated by one‐way ANOVA, and results were considered significant at *p* < .001. The Bonferroni test was used for post‐hoc comparisons when significance was determined by analysis of variance (*p < .001). The delivery rate was significantly increased in the group where the application phase was performed on the FBF compared to the group using only the toothbrush (TB) (*p* < .001). In contrast, there was no significant difference between FBB and TB. Between the FBF and FBB groups, there was no significant difference in the interproximal delivery rate depending on the presence or absence of the brushing phase (FBF + TB, FBB + TB)

On evaluation of interproximal toothpaste delivery rates by image analysis, the delivery rate was significantly increased in the group where the application phase was performed on the surface of the finger toothbrush (FBF) compared to the group using only the toothbrush (TB) (*p* < .001) (Figure 4). In contrast, there was no significant difference between the group where the application phase was performed on the back of the finger toothbrush (FBB) and TB. Although the delivery rates of TB and FBB were around 20%, FBF showed a remarkable difference of around 65%. Between the FBF and FBB groups, there was no significant difference in the interproximal delivery rate depending on the presence or absence of the brushing phase (FBF + TB, FBB + TB).

### 3.6 Interproximal model image analysis after application phase

**Figure 5:**
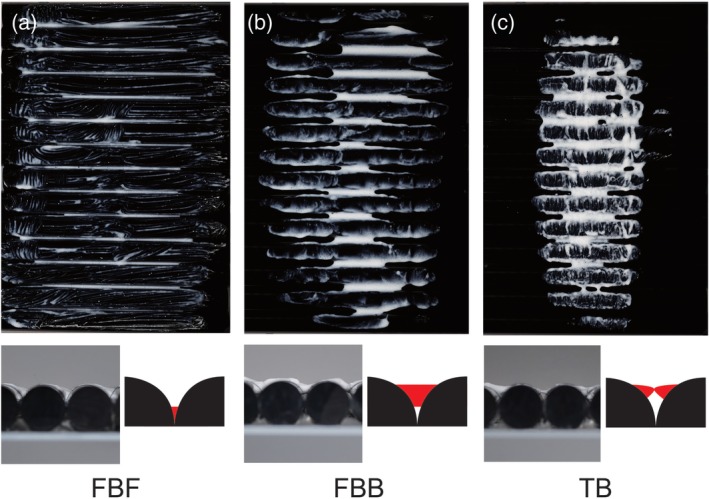
Interproximal model image analysis after application phase. Interproximal model: vertical picture and horizontal cross‐section image after the application phase. (a) FBF image, (b) FBB image, and (c) TB image. The side figure of horizontal cross‐section image is an enlarged view of the interproximal area. Black, acrylic model; red, toothpaste. FBB, finger brush back; FBF, finger brush front; TB, toothbrush

According to the interproximal model images of each group after the application phase, the toothpaste spread over the entire surface of the FBF model (Figure 5a). However, FBB and TB have a smaller area to which the toothpaste adheres compared with FBF (Figure 5b,c). On observing the horizontal cross‐section, the toothpaste was seen to fill the deep part of the groove in the FBF. However, the toothpaste adhered only to the upper part of the groove and appeared like a bridge in the FBB and TB (Figure 5a–c).

## 4 DISCUSSION

### 4.1 Changes in the physical properties of toothpaste by dilution

As the dilution ratio increased, the viscosity of the toothpaste decreased sharply, the *F* uptake decreased, and the dissolution rate increased. Although the fluoride concentration decreased from 950 ppm in the original solution (×1.00) to 760 ppm at ×1.25 dilution, the fluoride uptake tended to be higher at ×1.25 than that with the original solution (×1.00). It is considered that the decrease in the viscosity from 2,330 to 380 dPa·s facilitates the movement of the fluoride ion. In the case of an aqueous solution, since the fluoride concentration and uptake are proportional, it is considered that the amount of fluoride uptake is strongly influenced by the fluoride ion concentration when the viscosity is 380 dPa·s or less. This phenomenon is considered to be due to a general property of pastes (Figures 1 and 2). In the oral cavity, where dilution occurs continuously and unevenly, it is considered that the action of fluoride is greatly influenced not only by the concentration but also by the viscosity due to dilution. From this result, it was revealed that fluoride uptake was significantly reduced when the toothpaste was diluted by ×1.75 or more. Therefore, in order to improve the effectiveness of the fluoride containing toothpaste, it is considered important to deliver the toothpaste to high‐risk sites such as interproximal areas and pits and fissures at low dilution. Previous studies have reported that toothpastes are diluted about three times in 2 min in the oral cavity (Koga, Yamagishi, Takayanagi, Maeda, & Matsukubo, 2004). The fluoride uptake of a toothpaste diluted more than ×3.00 is considered to be low, and the caries preventive effect at the high‐risk interproximal areas is reduced compared with the original solution (×1.00) (Figure 1). The authors calculated the “expected effect index (*F*/*D*)” from the relationship between fluoride uptake (*F*) and dissolution rate (*D*) (Table 2). The expected effect index is an index for quantitatively evaluating the relationship between the toothpaste viscosity and the amount of residual toothpaste and fluoride uptake, which changes with dilution of the toothpaste. It decreased as the dilution ratio increased, and the original solution (×1.00) showed the highest value. Tooth surfaces to which a toothpaste is directly applied can be expected to be exposed to fluoride for a long time at a high concentration. Further, it is considered that the toothpaste applied to the tooth surface is gradually diluted with saliva and continues to release fluoride to the oral environment for a long time. Therefore, effective brushing methods for preventing dental caries need to deliver toothpaste to the interproximal areas and fissures, which are sites at high risk of dental caries in the state close to the stock solution. Moreover, it is thought that the device used with the brushing method and the selection of the toothbrush are important factors.

### 4.2 Delivery of toothpaste to the interproximal area

**Figure 6:**
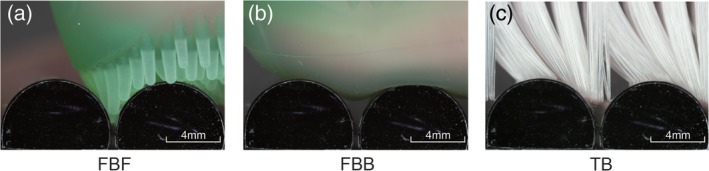
Horizontal cross‐section interproximal model image analysis. Interproximal model: horizontal cross‐section image. (a) FBF image, (b) FBB image, and (c) TB image. (a) It was shown that fed into the interdental area since the projection enters the interdental area while being deformed. (b) There is no structure that can enter between the teeth, so a large gap is created between the teeth when there is no projection. (c) The toothbrush was showed as control. FBB, finger brush back; FBF, finger brush front; TB, toothbrush

According to the experimental results, the FBB could application the most amount of toothpaste to the interproximal region compared with TB and FBF, but the delivery rate was not different from that observed with TB (*p* < .001) (Figures 3 and 4). This result indicates that the FBB can carry the toothpaste but cannot deliver it deep into the groove. The working surface of the FBB is flat, and it is excellent in terms of scrape off toothpaste by the edge of the groove. However, when the amount of toothpaste is large, the toothpaste can be delivered to the deepest part of the ditch by filling the entire ditch, but cannot be delivered to the deep part of the ditch when the amount of toothpaste decreases. From the image analysis results, it can be observed that the toothpaste did not reach the deepest part of the groove in the FBB (Figure 5b). Furthermore, the fact that the FBB is flat also means that it is impossible to spread the toothpaste once it has entered the groove. It is clear that the delivery rate is lower and that the toothpaste elongation is smaller compared to FBF from the images (Figures 3 and 5a,b). In contrast, in FBF, the amount of toothpaste applied on the model is the same as that with TB, but the delivery rate is significantly higher compared with TB and FBB (*p* < .001) (Figures 3 and 4). This result indicates that the FBF can push the toothpaste transferred to the groove into the deep part of the groove and extend it; further, it can be confirmed from the image that the toothpaste reached the deep part of the groove (Figure 5a). There is no clearance of toothpaste because the acting surface of the FBF has a small height and the width of the projections, and the projections fill most of the groove volume (Figure 6a). In other words, it is considered that the FBF can exert pressure while blocking the groove space to prevent toothpaste outflow.

There was no improvement in the delivery rate after the brushing phase in all groups (Figure 4). Our results indicate that it is difficult to push the undiluted toothpaste in the groove with a toothbrush. Therefore, it is important to insert the toothpaste deep into the groove at the stage of the application phase. In addition, followed brushing phase only FBB significantly reduced the amount of toothpaste (Figure 3). This is considered to be due to trapping of the toothpaste in the bristle tuft of the toothbrush in the brushing phase. In the case of FBB and TB, a large amount of toothpaste was present not at the deep part of the groove but at the shallow part (Figure 5b,c). It is assumed that the thin nylon tuft could not push the toothpaste in the shallow part into the deep part of the groove, and the toothpaste was trapped in the bristle tuft of the toothbrush. In the case of the FBF, there is no suitable bristle length for trapping the toothpaste as observed with the bristle tuft of a toothbrush, and the toothpaste can be inserted deep into the groove that is difficult to reach with the toothbrush bristle tip (Figure 4).

### 4.3 Instruments of PTD technique

According to Takayanagi et al., high‐risk areas such as areas between the teeth are difficult to reach with the tip of the toothbrush. Therefore, topical delivery of fluoride by the combined use of a toothbrush and dentifrice is stated to be an important factor in caries prevention (Takayanagi, 2006; Yamagishi, Suzuki, & Maeda, 2005). In addition, by making the head of the toothbrush convex or tapering, access to the interproximal part in polishing becomes high. It has been reported that the delivery of a toothpaste to the interproximal area varies depending on the toothbrush shape and processing (Yamagishi et al., 2005). For effective use of the PTD technique in self‐care, the choice of tools such as toothbrushes is considered to be an important point.

It is considered to be fed into the interdental area since the projection enters the interdental area while being deformed and can be in a form with a small gap (Figure 6a). In contrast, there is no structure that can enter between the teeth, so a large gap is created between the teeth when there is no projection (Figure 6b). The toothbrush can enter the interdental area, but the bristles are made of a harder nylon than silicone rubber. Therefore, it is considered that the interdental delivery rate is reduced because it cannot be deformed and brought into close contact (Figure 6c). Our results showed that products with the following conditions, bristles length (2–6 mm), width (0.5–3 mm), hardness (10–80: ISO 7619) and distance between bristles line (0.1–5 mm) are considered desirable in order to improve interdental deliverability. The best finger toothbrush construction needs to be considered because it varies with age and form of dentition. The commercially available products available for PTD technique are shown in Figure S4. We would like to find a toothbrush design that is effective for PTD technique, using toothbrushes with different hair forms and flocking patterns. We consider it desirable to perform flossing before the PTD method and remove plaque on the adjacent surface. However, it is better to avoid flossing after execution because it may remove the dentifrice remaining on the adjacent surface. One of the advantages of PTD is that it can enhance the effect of preventing caries without using floss. Floss itself has little evidence of caries prevention. In addition, it is difficult for children and elderly people to clean adjacent surfaces. Floss is disposable and not friendly to the global environment. The silicone finger brush used in PTD can withstand specifications for several years and has low economic and environmental impact.

### 4.4 Effective usage of toothpaste in PTD technique

In a study conducted by Yamagishi et al., it was clear that in order to effectively incorporate fluoride, it is necessary to use fluoride at 300 ppm or more for 2 min or more (Koga et al., 2004). Moreover, in order to satisfy this condition, it is desirable to use 1.0 g or more of toothpaste containing 1,000 ppm of fluoride (Koga, Yamagishi, Takayanagi, Maeda, & Matsukubo, 2007). Although a toothpaste should be delivered to the interproximal region with a toothbrush, many toothpastes penetrate between the bristle tufts of the toothbrush and cannot be delivered to interproximal area. Moreover, these toothpastes do not exert caries preventive effects. However, the loss of toothpaste can be reduced by delivering the toothbrush with a finger brush before brushing. This method is effective to reduce the decrease in fluoride concentration of the dentifrice in the oral cavity. In addition, the amount of toothpaste used is the same as that used with the conventional brushing method, and safety is also ensured. The PTD technique is an excellent method that can increase the caries preventive effect while using the same amount of toothpaste.

In order to improve the effectiveness of fluoride containing toothpastes, it is important to deliver the toothpaste at high‐risk sites such as the interproximal areas and pit clefts at low dilution. With the PTD technique, it is possible to achieve a high delivery rate of toothpaste with low dilution in the application phase, which can be expected to enhance the caries preventive effect without changing the amount and concentration of toothpaste. The finger brush is suitable for the PTD technique because it can close the groove space and application pressure while preventing the toothpaste from flowing out. The PTD technique is a brushing method that takes into account the effect of change in the physical properties and dilution of the fluoride‐containing toothpaste on the fluoride uptake. The PTD technique is considered to be effective for preventing dental caries in the interproximal area where the caries risk is high.

Although this study was a model experiment, it is considered that it will be necessary to verify the effect of the PTD technique on fluoride concentration in dental plaque and caries prevention in future. In addition, development of a toothbrush in consideration of its interaction with the toothpaste and reachability and development of a toothpaste having physical properties suitable for clearance delivery and high residual property are required.

## 5 CLINICAL RELEVANCE

### 5.1 Scientific rationale for study

We developed a method to improve the effect of fluoride on interproximal area, which focused on physical properties and functions of the toothpaste.

### 5.2 Principal findings

It was revealed that fluoride uptake was significantly reduced when the toothpaste was diluted by ×1.75 or more. PTD technique could apply more toothpaste to the interproximal areas compared using toothbrush alone.

### 5.3 Practical implications

It is important to deliver the toothpaste at high‐risk sites such as the interproximal areas and pit clefts at low dilution. The PTD technique with finger brush can be expected to enhance the caries preventive effect without changing the amount and concentration of toothpaste.

## CONFLICT OF INTEREST

The authors declare that there is no conflict of interest directly relevant to the content of this article.

## Supporting information

Appendix Figure S1 Image of Finger brush

Appendix Figure S2 Method of analyzing dissolution rate of toothpaste

Appendix Figure S3 Production of acrylic interproximal model and analyzing delivery rate of toothpaste

Appendix Figure S4 The commercially available products available for PTD technique
