# Supplementary figures and images for: Modified toothpaste application using prepared toothpaste delivering technique increases interproximal fluoride toothpaste delivery
Source: Clin Exp Dent Res. 2019 Dec 12;6(2):188–96. doi: 10.1002/cre2.268 (PMC7133740; doi:10.1002/cre2.268)

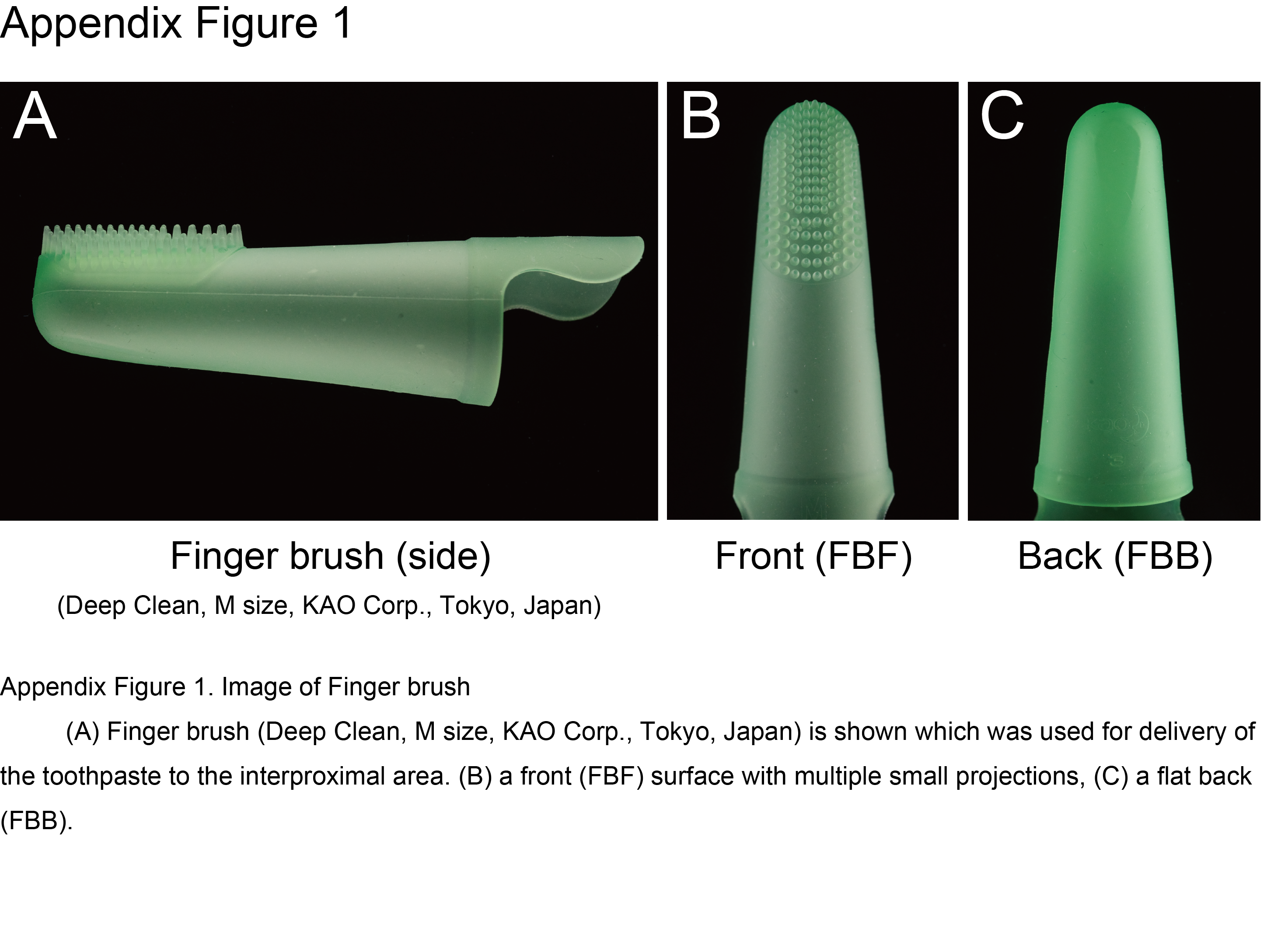

Supplement: Supplementary file 1 — Appendix Figure S1 Image of Finger brush [file CRE2-6-188-s001.tif]

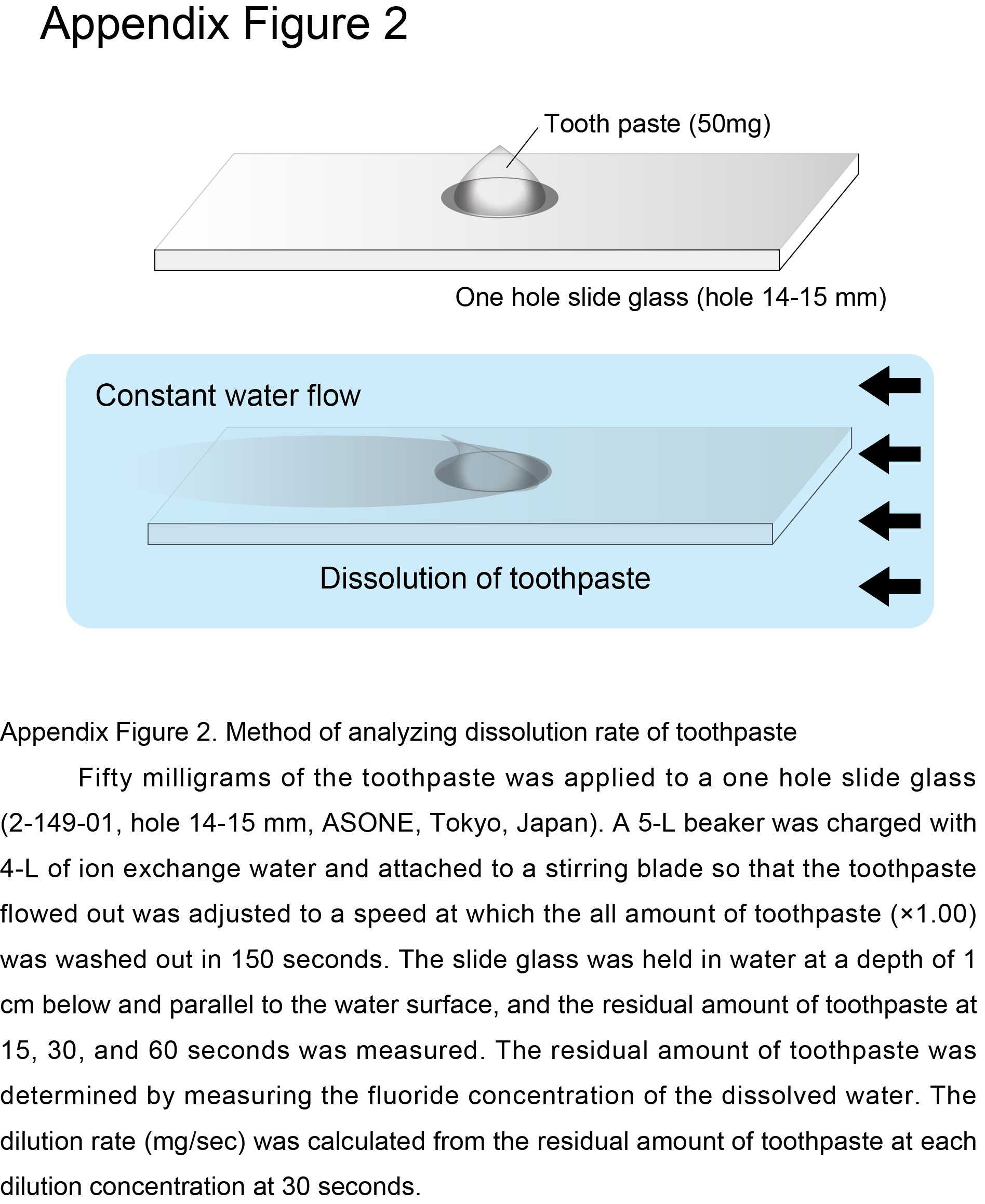

Supplement: Supplementary file 2 — Appendix Figure S2 Method of analyzing dissolution rate of toothpaste [file CRE2-6-188-s002.tif]

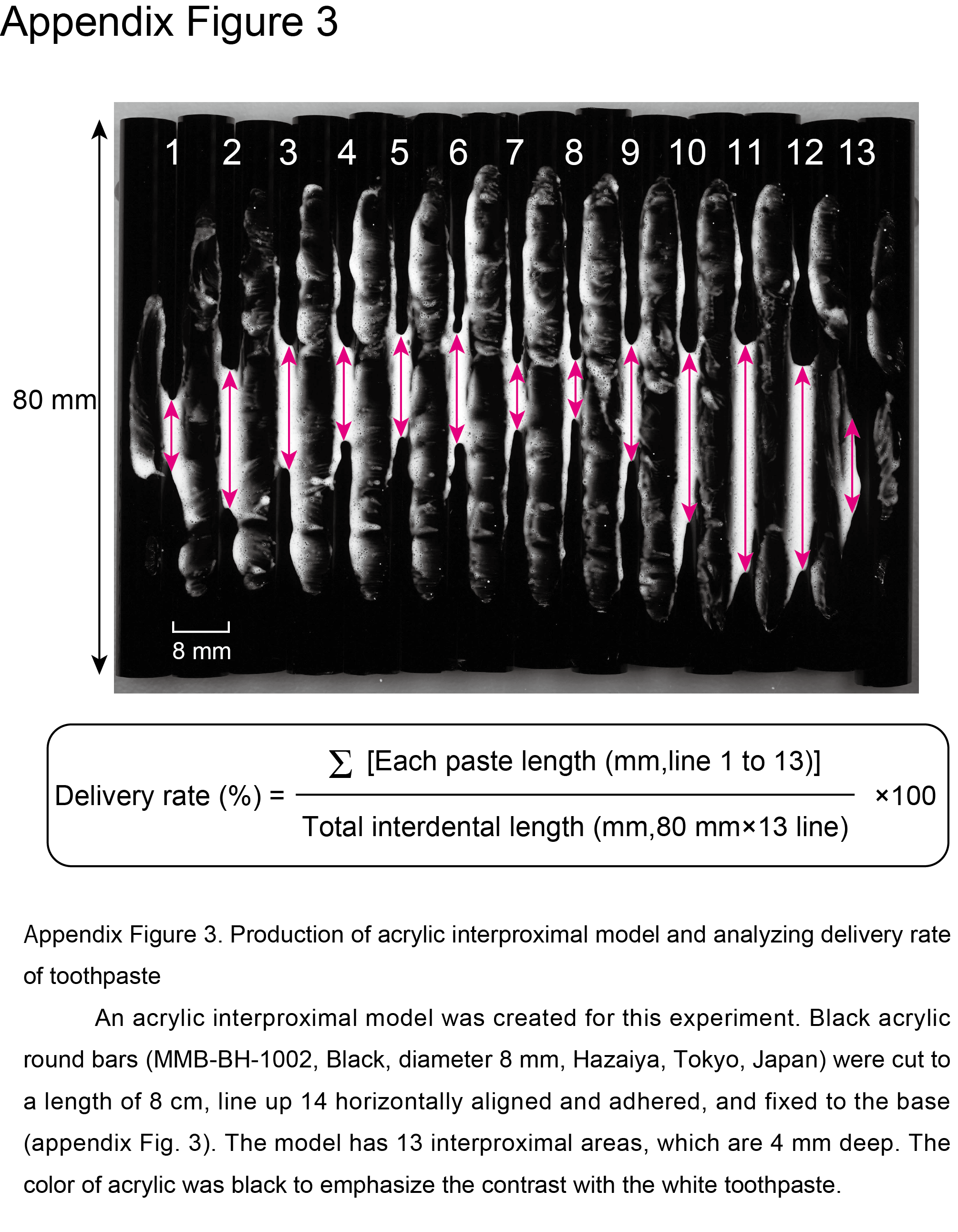

Supplement: Supplementary file 3 — Appendix Figure S3 Production of acrylic interproximal model and analyzing delivery rate of toothpaste [file CRE2-6-188-s003.tif]

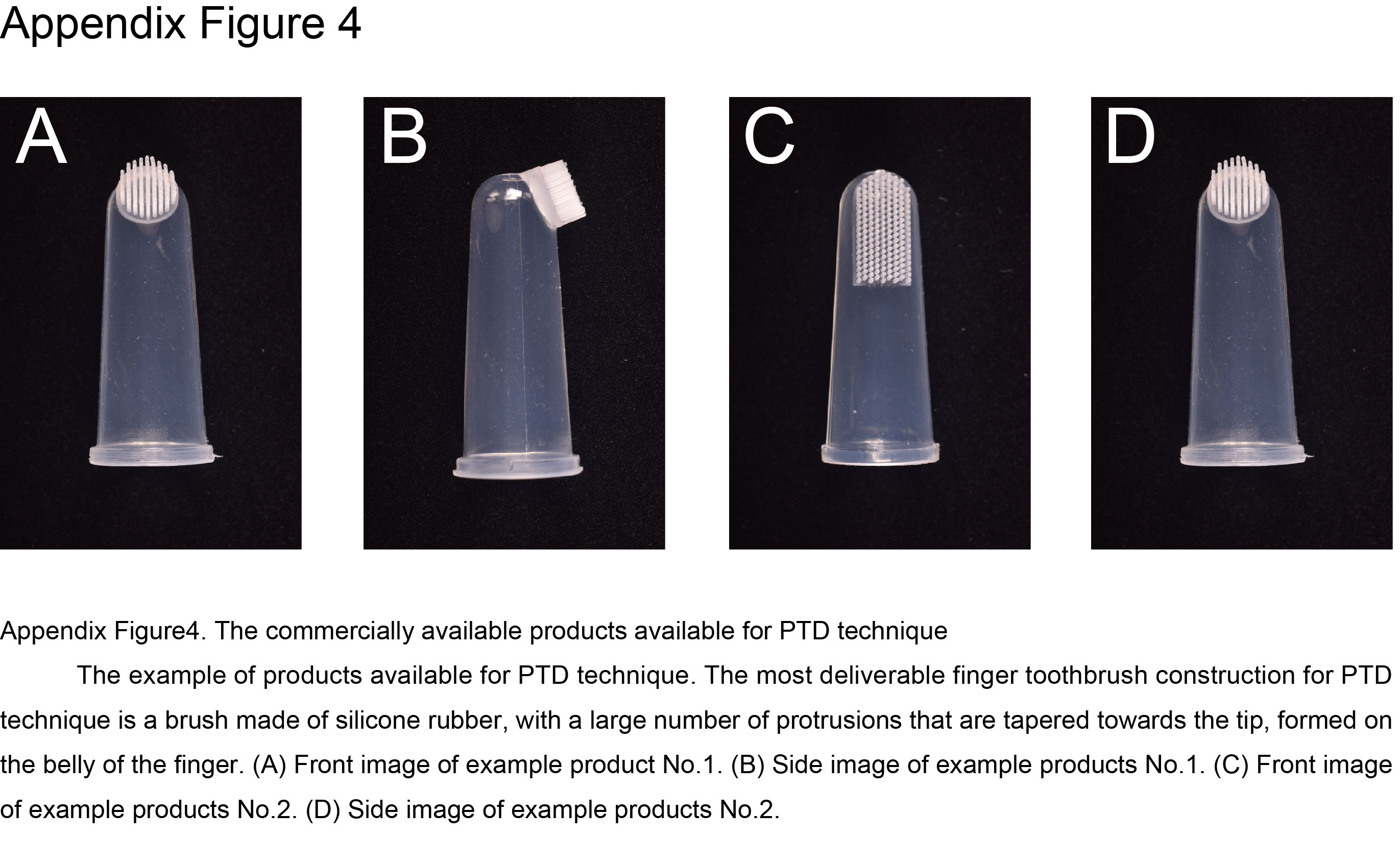

Supplement: Supplementary file 4 — Appendix Figure S4 The commercially available products available for PTD technique [file CRE2-6-188-s004.jpg]
